# Recalling Perceptions, Emotions, Behaviours, and Changes in Weight Status Among University Students After the Pandemic Experience

**DOI:** 10.3390/nu17193132

**Published:** 2025-09-30

**Authors:** Luciana Zaccagni, Stefania Toselli, Emanuela Gualdi-Russo

**Affiliations:** 1 Department of Neuroscience and Rehabilitation, Faculty of Medicine, Pharmacy and Prevention, University of Ferrara, 44121 Ferrara, Italy; luciana.zaccagni@unife.it (L.Z.); emanuela.gualdi@unife.it (E.G.-R.); 2 Center for Exercise Science and Sports, University of Ferrara, 44123 Ferrara, Italy; 3 Department for Life Quality Studies, University of Bologna, 47921 Rimini, Italy

**Keywords:** COVID-19, Sport Sciences students, sex, physical health, mental health, body mass index, physical activity

## Abstract

**Background/Objectives**: Overweight and obesity continue to increase globally, a trend that has been exacerbated by the pandemic. This retrospective study examines the impact of suspending sports activities during the pandemic on the physical and psychological well-being of young Italian adults engaged in sports, paying particular attention to their nutritional status. In particular, the study focused on sex-related differences in perceptions, lifestyle, and body mass index (BMI) changes. **Methods**: A cross-sectional online survey was conducted among 515 students enrolled in the Sport Sciences program. All the students were aged 18 years or older. Participants completed an 18-item questionnaire assessing their experiences during the pandemic on a five-point Likert scale and retrospectively reported changes in physical activity and body weight. **Results**: Overall, 38.3% of males and 43% of females reported that restrictions negatively affected their lives, with notable differences emerging in habits, behaviors, and perceptions between sexes. Multivariate regression analysis showed that current BMI was influenced by anthropometric characteristics and variables related to lockdown experiences, in both sexes. **Conclusions**: The current BMI of examined students was significantly influenced by changes in weight status during the pandemic, resulting from an increased sedentary lifestyle and changes in eating habits, especially among males. These findings highlight that the pandemic differentially affected the lifestyles and perceptions of physically active young adults, with sex-specific consequences for mental and physical health.

## 1. Introduction

Overweight and obesity, major contributors to morbidity and healthcare costs, are steadily increasing worldwide [1,2,3]. The recent COVID-19 outbreak, which severely affected populations across the globe with high morbidity and mortality rates, further exacerbated this trend. Social distancing measures adopted by governments to contain the spread of the virus also had profound mental and physical health implications, which are still not fully understood [4,5]. In particular, the pandemic fostered sedentary behaviors and unhealthy lifestyles, especially among young people, contributing to increased body mass index (BMI) and raising the risk of chronic diseases [6].

Although initial studies primarily focused on children [7,8], the pandemic’s consequences for adults have also received widespread attention [9,10,11]. An increased prevalence of obesity and metabolic risk has been linked to unhealthy behaviors characterized by reduced physical activity (PA) and an increased consumption of high-calorie “comfort foods” [10]. Alarmingly, several surveys suggest that pandemic-related weight gain may persist long after restrictions were lifted [12,13].

Early assessments, often based on heterogeneous questionnaires (some validated and some not, with different scopes and age groups), highlighted significant lifestyle changes compared to the pre-pandemic period [14,15]. These changes were particularly focused on dietary modifications [14].

The development of healthy lifestyle habits is strongly shaped during late adolescence and early adulthood [16]. While active behaviors during childhood and adolescence may be carried into adult life [17], a decline in PA is often reported upon entering university, with 40–50% of students becoming physically inactive [18], although with clear differences between nations depending on both cultures and education [16]. Students enrolled in Sport Sciences programs, in particular, are expected to maintain higher PA levels due to their vocation and curriculum. However, restrictions and suspension of organized sport during the pandemic may have disproportionately affected these students, who typically pursue an active lifestyle. Indeed, studies on athletes (Badminton and Soccer players) have shown that prolonged training suspension resulted in unfavorable changes to body composition, including an increase in fat mass (FM) and a reduction in fat-free mass (FFM) [19,20]. A recent meta-analysis of 24 studies on the topic found that, while athletes managed to prevent significant changes in body composition, they did experience significant increases in total body weight [21].

In Italy, all sports events and activities were suspended in March 2020, with gradual resumption only beginning in late April 2021 [22]. These restrictions were implemented alongside broader closures of schools, universities, workplaces, and recreational facilities, with online teaching adopted nationwide. Although the World Health Organization declared the official end of the global health emergency on 5 May 2023, hybrid online learning formats continue to be used in Italian universities.

In Europe, several studies further confirmed the negative effects of COVID-19 restrictions on body composition and PA among young adults, with notable sex-related differences [23,24,25,26,27]. The lockdown also had a profound psychological impact, increasing fear of infection, anxiety, and depression [28,29,30,31]. Declines in well-being were particularly marked among women, young adults, individuals with pre-existing mental health conditions, and those in low-income settings [32,33,34,35,36]. Although the negative effects on mental health appear to have diminished over time [37], persistent symptoms have been reported among COVID-19 survivors, including sleep disturbances, anxiety, depression, and headaches [38]. Even now, some time after the pandemic ended, little is still known about its long-term effects or whether any post-traumatic disorders have developed as a result [39]. Therefore, further research is needed to improve our understanding of the long-term effects of the pandemic and to identify effective coping strategies, such as exercise and social support, that may mitigate the impact of future health crises. Building on earlier research conducted among adolescents [40], this study extends the investigation to young adults enrolled in Sport Sciences programs at two Italian universities. Despite existing studies on the behaviour of undergraduate students before and after the pandemic [16,30,41,42,43], evidence addressing the mental and physical health of Sport Sciences students specifically remains limited, particularly from a retrospective perspective. This study adopted a retrospective approach to integrate previous methodological approaches into a questionnaire tailored to the sporting population, assessing both recollections of lived experiences and concerns about future pandemics over time. In particular, the present study aims to investigate potential sex differences in physical and mental health among Sport Sciences students following the lockdown. Using a newly developed and validated instrument, we intend to examine any changes in body weight and PA habits, perceptions of lifestyle modifications, and concerns about future epidemics. Given the growing concern about obesity and health, our focus will be on the factors that may have influenced the current BMI of the students examined, analysed separately by sex.

## 2. Materials and Methods

### 2.1. Research Design, Participants

**Figure A1:**
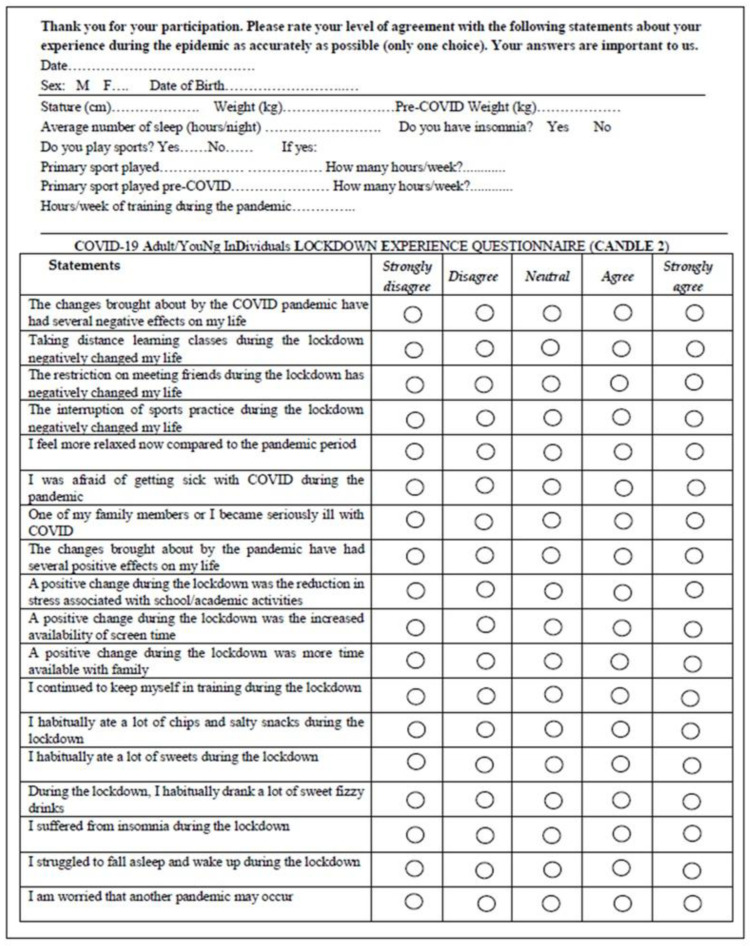
Data collection sheet.

This study employed a cross-sectional design to describe the target population at a single point in time. Based on the literature concerning COVID-19-related stress in adolescents and adults [44,45,46,47] and the influence of pandemic-related lifestyle changes on mental health and behavior, we developed a new questionnaire. To enhance its content validity, three experts in sports, nutrition, and psychological sciences reviewed the preliminary version and suggested modifications. Following their recommendations, three redundant statements were removed, reducing the total from 21 to 18. A short pilot test was subsequently conducted with family members and peers within the target age group (n = 13), leading to additional minor revisions aimed at improving clarity. The final version of the instrument is reported in Table 1, and the English translation is provided in Appendix A (Figure A1). Responses were collected using a 5-point bipolar Likert scale: 1 = Strongly Disagree, 2 = Disagree, 3 = Neutral, 4 = Agree, 5 = Strongly Agree.

**Figure 1:**
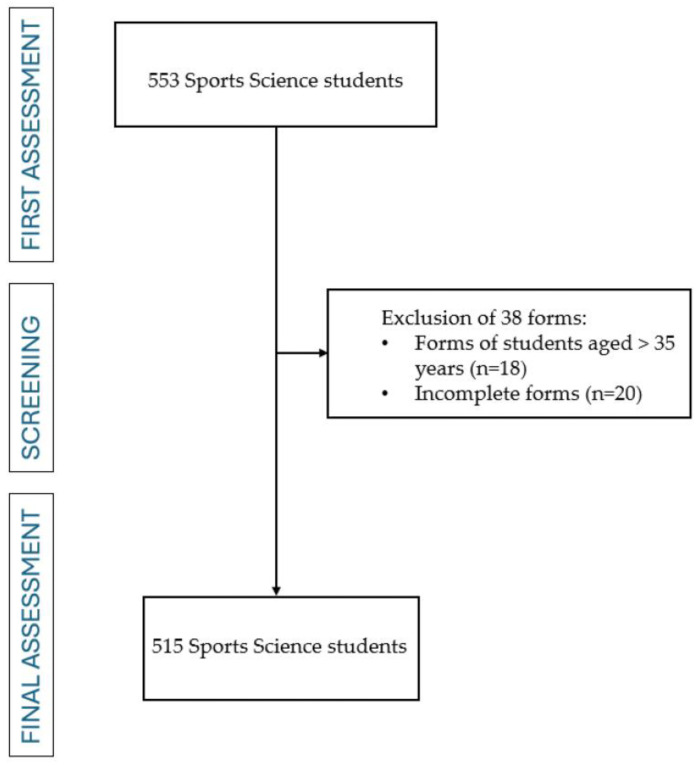
Flow diagram showing the participant selection and exclusion process.

The new instrument was developed by adapting and expanding an existing tool, the CANDLE questionnaire, originally designed by us for Italian children and adolescents [40]. While the earlier version consisted of 11 simple questions on experiences during the first national lockdown, the revised tool (CANDLE 2: COVID-19 Adult/YouNg InDividuals LOCKDOWN EXPERIENCE QUESTIONNAIRE) was more detailed and targeted young adults (18–35 years) three years after the pandemic. From 1 December 2024, to 28 February 2025, all Sport Sciences students from the universities of Ferrara and Bologna (Northern Italy) were invited to complete a web-based survey via their institutional learning platforms. Recruitment was supported through e-mail announcements and notifications during tutorials. Participation was voluntary, and participants were guaranteed anonymity and confidentiality. Figure 1 summarizes the steps of participant inclusion and exclusion.

A total of 553 responses were initially received. After excluding 18 forms from students over 35 years of age and 20 incomplete submissions, the final sample comprised 515 Sport Sciences students (379 males and 136 females), with a mean age of 23.0 ± 1.9 years. This sample exceeded the minimum required size calculated through an a priori power analysis conducted with G*Power (version 3.1.9.6; Universität Kiel, Kiel, Germany). Assuming an 80% power, a medium effect size, and α = 0.05, the minimum required sample for primary analyses (sex comparisons) was 128 participants for quantitative traits (one-way ANOVA) and 122 for qualitative traits (chi-square test), respectively, following Andrade [48].

The questionnaire was administered via Google Forms and preceded by a web page containing a detailed description of the study, along with information on voluntary participation. By filling out and returning the questionnaire, students agreed to take part in the survey. All responses were stored anonymously, linked only to the e-mail account used for access.

The study protocol was approved by the Ethics Committee of Area Vasta Emilia Centrale (Ethical Approval No. VT2022; date of approval: 15 February 2023).

### 2.2. Procedures

Participants provided socio-demographic and anthropometric data, including sex, age, stature, current weight, pre-COVID weight, sleep patterns, and PA habits before, during, and after the lockdown. They then completed the CANDLE 2 questionnaire, which retrospectively assessed their emotional and behavioral recall of the COVID-19 lockdown.

To evaluate test–retest reliability, we invited 50 randomly selected participants to complete the questionnaire again one month later. Thirty-six students (response rate: 72%) agreed to participate, of whom 35 provided complete data. Then, the same sample was used to validate regression models and evaluate accuracy, precision, and estimated bias over time.

Body mass index (BMI) was calculated as weight (kg) divided by stature squared (m^2^) and used to classify participants’ weight status as underweight, normal weight, overweight, or obese. When substantial inconsistencies in the quantitative variables were detected during data screening, participants were contacted via e-mail to verify their data.

### 2.3. Statistical Analysis

Data are presented as means, standard deviations, and frequencies. The Kolmogorov–Smirnov test was applied to assess normality.

Internal consistency of the CANDLE 2 questionnaire was evaluated using Cronbach’s alpha, and test–retest reliability was analyzed in the subsample of repeated responses.

Descriptive statistics were applied to self-reported anthropometric data and PA habits. Differences between sexes were examined using chi-square tests for qualitative traits and one-way ANOVA with Bonferroni correction for quantitative traits.

To identify predictors of current BMI, backward multiple regression analyses were conducted separately by sex. This procedure retained the significant predictors and removed the non-significant ones (*p* < 0.05), using R^2^ as the selection criterion. Two additional regression models were performed on the subsample with repeated measures. Model fit was evaluated using R^2^ and F-statistics, while precision and accuracy were assessed through the Concordance Correlation Coefficient [49]. Cross-validation techniques were applied to confirm robustness and avoid overfitting. To assess the agreement between the two measurement methods, we used the Bland–Altman plot, which displays the difference between the measurements against their mean, allowing identification of any systematic bias and the limits of agreement [50].

All statistical analyses were performed with STATISTICA for Windows, Version 11.0 (StatSoft Inc., Tulsa, OK, USA). Results were considered statistically significant at *p* < 0.05.

## 3. Results

The internal consistency of the CANDLE 2 questionnaire was acceptable, with a Cronbach’s alpha of 0.79. Test–retest analysis confirmed good reliability, with correlation coefficients ranging from 0.78 to 0.97 (Table 2).

Anthropometric characteristics, sports practice, and sleep habits by sex are reported in Table 3. As expected, male students were significantly taller and heavier than females and showed higher BMI values. Both sexes reported increases in weight and BMI compared to the pre-COVID period, although changes were more pronounced among males. Specifically, male students exhibited significant increases in both weight and BMI (*p* < 0.001), while in female students, differences approached significance (weight: *p* = 0.064; BMI: *p* = 0.051).

Weight increased in 70.1% of males and 43.7% of females, remained stable in 10.6% and 20.6%, and decreased in 19.1% and 35.7%, respectively. Sex differences in the distribution of weight changes were highly significant (χ^2^ = 27.14; *df* = 2; *p* < 0.001).

Regarding sports practice, males reported significantly more weekly hours of training than females. However, in the pre-COVID period, both sexes devoted similar amounts of time to sport, with higher values than those currently observed, particularly among females. During lockdown, weekly training hours declined in both sexes, without significant differences between them. In terms of sleep, the average duration did not differ; however, females reported a higher prevalence of insomnia.

Analysis of weight status (Table 4) revealed significant changes among males, with an increased prevalence of being overweight and a decreased prevalence of being underweight (χ^2^ = 17.04; *df* = 3; *p* < 0.001). No significant changes were observed in females.

Between-sex comparisons showed no differences in pre-COVID weight status (χ^2^ = 6.13; *df* = 3; *p* = 0.106), but significant differences in the current period (χ^2^ = 28.83; *df* = 3; *p* < 0.001), with females more likely to be underweight or normal weight, and males more likely to be overweight or obese.

Table 5 presents the prevalence of agreement with questionnaire statements by sex. Significant sex differences were observed for S6, S9, S10, S15, S16, and S18. Female students more frequently reported fear of contracting the virus (S6, *p* = 0.011) and insomnia during lockdown (S16, *p* = 0.048). Male students were more likely to adopt a neutral position regarding reduced school-related stress (S9, *p* = 0.017). The perception of increased screen time as a positive change (S10) differed markedly between sexes, with more males than females agreeing (*p* < 0.001). Males also reported higher consumption of sugary drinks (S15, *p* = 0.013). Finally, the majority of female students agreed on concerns regarding a new pandemic (S18, *p* < 0.001).

Common trends (i.e., no sex differences) included the negative impact of pandemic restrictions (S1), distance learning (S2), reduced social contact (S3), and interruption of sports training (S4). Most students reported positive experiences during lockdown, such as spending more time with family (S11) and continuing to exercise (S12). Few reported positive overall effects of the pandemic (S8), and most denied increased comfort food consumption (S14).

Multiple regression analyses (Table 6) revealed that pre-pandemic weight status and weight change were significant predictors of current BMI in both sexes. Additionally, statement S7 (absence of severe illness among family members/participants) was positively associated with BMI. In males, interruption of sports training (S4) was a further predictor, while in females, increased family time (S11) showed a positive association. Models evaluating the influence of anthropometric traits and pandemic experience on BMI were significant (*p* < 0.001), with an R^2^ value of 64% for both sexes.

**Figure 2:**
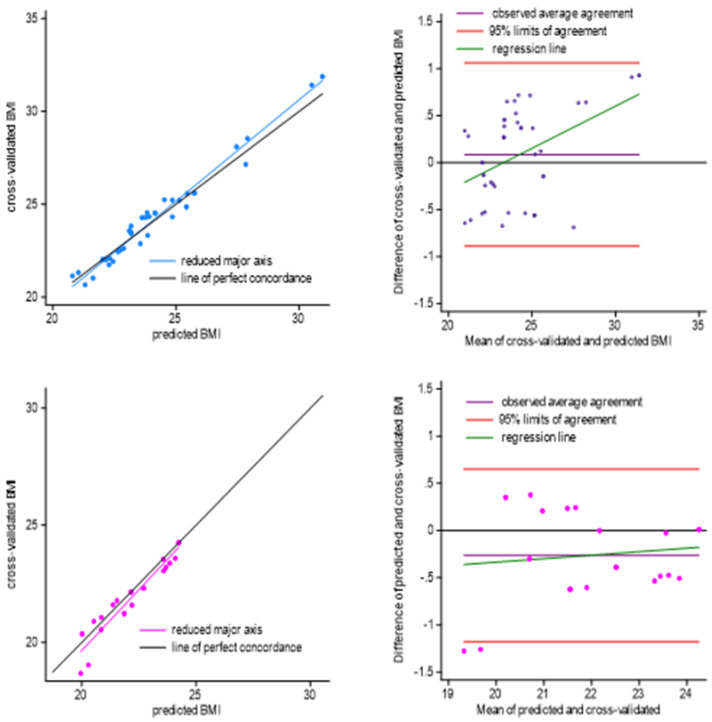
Regression line showing predicted BMI and cross-validated BMI values (on the **left**), and a Bland–Altman plot comparing the differences between predicted and cross-validated BMI (on the **right**). Males are displayed at the top and females at the bottom.

Repeated measures analysis, assessed on a sub-sample of male and female participants, confirmed the model robustness (Figure 2), with different fits between sexes. For females, at the first measurement, the model explains an R^2^ of 0.54, while a reduced explanation appears at cross-validation (R^2^ = 0.48). The degree of agreement between predicted BMI at pre and post COVID is 93% (95% CI = 0.88 to 0.99), with a mean 2% BMI underestimation. The precision between predicted and cross-validated models is 95%, shown by the correlation coefficient between reduced major axis and line of perfect concordance (Figure 2, pictures at bottom). The 95% limits of agreement by the Bland & Altman plot varied from −1.18 to +0.65, with a deviation from perfect concordance by −0.26 ± 0.47. Two participants lie outside the 95% estimated limits of agreement. Estimation bias from predicted to cross-validated BMI weakly increased by ~1% (~7 to 8%). In terms of goodness of fit, the prediction variability explained by the first model is 2% (R^2^ = 81%, F_(5, 17)_ = 13.66, *p* < 0.001), less effective than cross-validated BMI (R^2^ = 83%, F_(5, 17)_ = 17.43, *p* < 0.001).

Regarding males’ cross-validation assessment, agreement between test and retest validation is almost perfect (98%, 95% CI: 0.97, 0.99). The correlation coefficient between predicted and cross-validated lines is 0.99 (precision) with a similar degree of accuracy (99%, Figure 2, blue scatters). No participant lies outside the Bland & Altman plot 95% lines of agreement, estimated within −0.89 to +1.06, with an average deviation from perfect concordance of 0.09 ± 0.50. The mean difference between two models increases as their estimated difference increases, showing that the great models’ variation is due to participants with the highest BMI. At the first assessment, the estimated bias was 6.7% while it decreased to 5.7% at time two, with a mean overestimation of observed values.

## 4. Discussion

The COVID-19 pandemic profoundly disrupted daily life, affecting not only those directly infected with the disease but also the general population due to widespread restrictions. Since then, numerous studies have examined the physical and psychological consequences of this unprecedented event [7,10,11]. Addressing a gap in the literature, our study focused on young adult athletes—specifically Italian Sport Sciences students—who were forced to interrupt organized sport activities during lockdown, relying instead on individual or home-based exercise. Using the newly developed and validated CANDLE 2 questionnaire, we retrospectively assessed anthropometric changes, health-related behaviors, and perceptions three years after the first lockdown, investigating their association with current BMI.

Two methodological considerations deserve mention. First, recall bias may have influenced participants’ perceptions, as individuals tend to reconstruct past experiences in a more positive light (“positivity phenomenon”) [51,52,53,54]. Therefore, given the time that has elapsed since the lockdown and the administration of the questionnaire, we cannot rule out the possibility that this bias has influenced participants’ memories, particularly concerning behavioural changes such as perceived stress. Second, although based on self-reported anthropometry, the data can be considered reliable. This method is widely used as it is less labour-intensive, time-consuming, and costly than objective measures. Furthermore, a recent systematic review confirmed the validity and reliability of self-reported weight, stature, and BMI measurements given the high correlations consistently documented between self-reported and measured data (>0.9) [55]. Therefore, we believe that we can use self-reported anthropometric data with confidence.

To achieve the aims of this study, a new questionnaire was developed and validated. The CANDLE 2 questionnaire showed satisfactory reliability and proved effective in capturing both shared and sex-specific experiences of the pandemic. Both males and females reported the negative consequences of lockdown—social isolation, suspension of sport activities, and increased sedentary time—while positively valuing closer family ties. However, distinct differences emerged: female students were more prone to fear of contagion, concern for future pandemics, and sleep disturbances, whereas male students reported greater appreciation of increased screen time and higher consumption of sugary drinks. This increase in the intake of sugary products may be linked to boredom, emotional regulation, or the lack of structured routines during lockdown, which has been widely reported in the literature as a common response to stressful or monotonous environments. However, only some local studies in Italy suggest an increase in sugary drinks in specific subpopulations, such as obese children [56], while there is no robust national-level data to confirm this trend. Excessive consumption of sugar-sweetened beverages may have adverse effects on both physical and mental health, as observed in Chinese students aged 19–22 [57]. The increase in obesity during lockdown may be due to lifestyle changes such as reduced PA and increased consumption of these beverages. Although our data on dietary habits were limited, these trends are consistent with international findings suggesting gender-based differences in nutritional coping behaviors during periods of crisis. In particular, emotional eating—defined as (over)eating in response to negative emotions—has been shown to increase during the COVID-19 pandemic. McAtamney et al. [58] found that individuals who reported changes in their eating behavior during lockdown also experienced higher levels of depression. Furthermore, it was found that difficulties in identifying and describing emotions (i.e., alexithymia) indirectly predict emotional eating through emotional dysregulation. This evidence suggests how negative emotional states and impaired emotional awareness can contribute to maladaptive eating patterns, particularly in contexts of prolonged stress and isolation, such as those experienced during pandemic-related lockdowns. In addition, these findings align with international evidence indicating that females experience stronger psychological distress and sleep disruption [59,60,61], while males are more affected by the interruption to sport [59].

Anthropometric outcomes also revealed sex-specific trends. Males gained more weight than females (+3.5 vs. <1 kg), resulting in a significant BMI increase, with a higher prevalence of overweight/obesity in the post-COVID period. These findings are consistent with longitudinal studies showing weight gain and rising obesity prevalence during the pandemic [62]. As in other populations, it is likely that unhealthy behaviours, including altered sleep and diet, contributed to these changes [61,62,63]. Comparisons with the earlier CANDLE study on adolescents [40] showed both similarities (e.g., positive judgment of family time, negative impact of social deprivation, more frequent female sleep disturbances) and differences (e.g., less evidence of comfort food use among male university students, except for sugary drinks).

PA declined during lockdown but partially recovered afterward, in line with previous reports in European and Italian students [16,46,64,65]. Sport Sciences students, however, appeared more resilient in maintaining PA habits than peers in other university courses, reflecting their health-related knowledge and motivation. Nevertheless, males continued to report more frequent and longer training sessions, confirming well-documented sex gaps in PA [66,67].

Regression analyses identified pre-COVID weight status and weight change as the strongest predictors of current BMI. Additional associations emerged: absence of severe COVID-19 cases in the family was linked to higher BMI, possibly reflecting greater psychological stability and regular lifestyles; among females, more time spent with family also predicted higher BMI, perhaps due to shared eating patterns, which may have contributed to higher caloric intake, especially if family gatherings became a central part of daily life. During lockdown, spending extended time with family could have led to more frequent communal meals and snacking occasions, often characterized by less structured eating schedules. This environment may encourage increased consumption of calorie-dense foods, either as comfort or social bonding practices. Such changes in dietary habits, combined with reduced opportunities for PA, might explain the observed association between time spent with family and higher BMI among females. The models explained approximately 64% of the variance in BMI for both sexes, indicating a robust predictive capacity.

This study has several strengths, including the development and validation of a novel tool for assessing COVID-19-related lifestyle and perception changes in physically active young adults. The same procedure with expert consultation, subsequent adaptation of the questionnaire, and subsequent validation helped to strengthen the study. Another strength is the involvement of physically active young adults. The analysis of this particular sample enabled us to derive information about individuals who undoubtedly suffered more from PA suspension during lockdown. However, the following limitations must be acknowledged: the dimensions explaining the relationships between the multiple items of the questionnaire were not explored using statistical procedures. Furthermore, reliance on retrospective self-reports, sex imbalance in the sample (males > 70%), and restriction to a single population limit generalizability. Future studies may analyze the results of administering the questionnaire to different, numerically sex-balanced samples.

## 5. Conclusions

The newly developed CANDLE 2 questionnaire, conceived as an extension of the original CANDLE instrument for children and adolescents, represents a valid and user-friendly tool for assessing lifestyle changes among young adults during the COVID-19 pandemic. Its standardized 5-point Likert scale format and concise structure ensured ease of administration while yielding reliable insights into behavioral and perceptual shifts compared with pre-pandemic habits.

Despite the inherent limitations of self-reported and retrospective data, the study highlights significant sex-related differences in the impact of the pandemic. Female students reported higher levels of anxiety and sleep disturbances, whereas males exhibited greater changes in weight and BMI, likely associated with unhealthy eating behaviors and increased screen time. Multiple regression analysis further underscored the role of pre-pandemic weight status, weight variations, and specific lived experiences in shaping current BMI outcomes.

Overall, the health risks in this cohort of Sport Science students appeared relatively limited, possibly mitigated by their active lifestyle and awareness of healthy habits. Nevertheless, the emergence of sex-specific vulnerabilities emphasizes the need for targeted, sex-sensitive interventions to safeguard both mental and physical health during and after public health crises.

The CANDLE 2 questionnaire has proven to be a reliable tool for assessing the impact of COVID-19 on lifestyle-related behaviours, with potential value for public health researchers in identifying these changes at the community level and developing strategies to promote corrective behaviours and inform public health recommendations. Promoting education on active and healthy lifestyles remains crucial to counteract sedentary behaviour and unhealthy food consumption during periods of isolation. Future research should examine the applicability of CANDLE 2 across broader populations, including students from other academic disciplines and educational levels, to validate its generalizability.

## Figures and Tables

**Table 1:** CANDLE 2: List of statements.

Statements
S1. The changes brought about by the COVID pandemic have had several negative effects on my life.
S2. Taking distance learning classes during the lockdown negatively changed my life.
S3. The restriction on meeting friends during the lockdown has negatively changed my life.
S4. The interruption of sports practice during the lockdown negatively changed my life.
S5. I feel more relaxed now compared to the pandemic period.
S6. I was afraid of getting sick with COVID during the pandemic.
S7. One of my family members or I became seriously ill with COVID.
S8. The changes brought about by the pandemic have had several positive effects on my life.
S9. A positive change during the lockdown was the reduction in stress associated with school/academic activities.
S10. A positive change during the lockdown was the increased availability of screen time.
S11. A positive change during the lockdown was more time available with family.
S12. I continued to keep myself in training during the lockdown.
S13. I habitually ate a lot of chips and salty snacks during the lockdown.
S14. I habitually ate a lot of sweets during the lockdown.
S15. During the lockdown, I habitually drank a lot of sweet fizzy drinks.
S16. I suffered from insomnia during the lockdown.
S17. I struggled to fall asleep and wake up during the lockdown.
S18. I am worried that another pandemic may occur.

**Table 2:** Test–retest analysis for CANDLE 2 statements.

Statement	Correlation Value	*p*-Value
S1	0.89	<0.001
S2	0.97	<0.001
S3	0.93	<0.001
S4	0.94	<0.001
S5	0.93	<0.001
S6	0.82	<0.001
S7	0.78	<0.001
S8	0.95	<0.001
S9	0.84	<0.001
S10	0.93	<0.001
S11	0.93	<0.001
S12	0.82	<0.001
S13	0.85	<0.001
S14	0.97	<0.001
S15	0.80	<0.001
S16	0.97	<0.001
S17	0.91	<0.001
S18	0.90	<0.001

**Table 3:** Comparison between sexes (males: n = 379; females: n = 136) for anthropometric traits, information on sports practice, and sleep.

Variables	Males		Females		*F*_(1, 514)_ χ^2^_(1)_	*p*-Value
Age (years)	23.06	2.01	23.00	1.61	0.11	0.744 ^a^
*Anthropometric traits*						
Weight (kg)	76.03	9.13	58.54	8.25	380.26	<0.001 ^a^
Stature (cm)	178.82	6.82	164.53	6.40	449.66	<0.001 ^a^
BMI (kg/m^2^)	23.77	2.43	21.63	2.72	71.20	<0.001 ^a^
Pre-COVID Weight (kg)	72.79	10.07	57.64	7.89	234.16	<0.001 ^a^
Pre-COVID BMI (kg/m^2^)	22.70	2.84	21.32	2.54	22.97	<0.001 ^a^
Weight change (kg)	3.49	7.23	0.76	4.55	15.78	<0.001 ^a^
*Sports participation*						
Actual sports practice (%)	92.3		86.0		4.13	0.030 ^b^
Actual sport amount (h/week)	7.5	3.8	6.5	4.0	10.12	0.002 ^a^
PA during the COVID (h/week)	5.7	4.1	5.4	4.3	0.63	0.429 ^a^
Pre-COVID sport amount (h/week)	8.4	8.0	8.0	5.5	0.11	0.743 ^a^
*Sleep duration and disorders*						
Occurrence of insomnia (%)	9.8		16.9		5.32	0.025 ^b^
Sleep duration (hours/night)	7.46	5.02	7.33	0.90	1.54	0.215 ^a^

^a^ Bonferroni correction; ^b^ chi-square test.

**Table 4:** Comparison between pre-COVID and current weight status in males and females.

Weight Status	Males		*p*-Value	Females		*p*-Value
	Pre-COVID %	Current %		Pre-COVID %	Current %	
Underweight	5.3	0.8	<0.001	8.7	8.2	0.969
Normal weight	76.9	74.3		82.5	80.6	
Overweight	16.6	23.0		8.7	10.4	
Obese	1.1	1.9		0.0	0.7	

**Table 5:** Percent frequency distribution of the level of agreement from 1 (Strongly Disagree) to 5 (Strongly Agree) with 18 statements in males (*n* = 379) and females (*n* = 136).

Statements	Males					Females					*p*-Value
	1	2	3	4	5	1	2	3	4	5	
S1	3.7	20.6	37.2	27.2	11.1	8.1	15.4	33.8	33.8	8.8	0.1018
S2	8.2	23.2	34.3	24.8	9.5	8.8	29.4	24.3	27.69	9.6	0.2709
S3	4.7	10.6	25.1	41.4	18.2	5.1	15.4	22.8	37.5	19.1	0.6112
S4	3.7	9.8	16.4	41.7	28.5	4.4	16.2	22.1	37.5	19.9	0.0521
S5	5.5	19.0	37.2	26.9	11.3	4.4	28.7	28.7	27.2	11.0	0.1533
S6	12.1	24.8	24.3	29.8	9.0	8.8	23.5	16.2	32.4	19.1	0.0110
S7	23.5	43.0	8.7	19.8	5.0	18.4	42.6	11.8	18.4	8.8	0.3219
S8	7.7	19.3	44.1	25.9	3.2	5.9	26.5	40.4	23.5	3.7	0.4790
S9	6.1	14.0	26.69	39.3	13.7	7.4	24.3	16.9	34.6	16.9	0.0173
S10	14.0	34.0	25.6	19.5	6.9	22.8	46.3	22.1	7.4	1.5	0.0001
S11	2.4	4.5	19.3	49.1	24.8	0.7	5.9	14.0	47.8	31.6	0.2735
S12	5.5	11.1	9.2	45.9	28.2	0.7	12.5	9.6	47.8	29.4	0.2208
S13	16.9	40.4	22.4	16.4	4.0	22.1	41.9	18.4	16.2	1.5	0.3835
S14	16.9	36.7	26.1	16.9	3.4	18.4	38.2	18.4	24.9	0.7	0.0828
S15	21.4	43.0	20.3	12.7	2.6	32.4	44.9	16.9	4.4	1.5	0.0126
S16	24.3	46.2	12.4	12.4	4.7	15.4	44.1	16.2	20.6	3.7	0.0481
S17	20.8	43.3	12.9	17.7	5.3	12.5	42.2	14.7	25.0	6.6	0.1290
S18	13.8	23.2	37.2	20.8	5.5	3.7	16.2	33.1	36.0	11.0	<0.0001

**Table 6:** Predictors of BMI by multiple backward regression analysis in males and females.

Predictor Variables	Males			Females		
	β	*p*-Value	VIF	β	*p*-Value	VIF
Weight change	0.727	<0.001	1.407	0.469	<0.001	1.041
S4	0.070	0.039	1.006			
S7	−0.089	0.009	1.007	−0.132	0.026	1.024
S11				0.130	0.030	1.053
Weight status pre-COVID *						
- Underweight	−0.666	<0.001	1.375			
- Normal weight	−0.449	<0.001	1.680	−0.161	0.011	1.153
- Overweight	0.258	<0.001	1.515	0.719	<0.001	1.139
R^2^		0.643			0.660	
R^2^ adjusted		0.636			0.643	
*p-value*		<0.001			<0.001	

* Reference category: obese in males, underweight in females.

## Data Availability

Data is available upon request to the authors due to ethical restrictions regarding participants’ privacy. The CANDLE2 questionnaire is freely available for use in research: no permission is required; however, appropriate citation of this article is requested.

## Abbreviations

The following abbreviations are used in this manuscript:

COVID-19	COronaVIrus Disease of 2019
PA	Physical Activity
CANDLE 2	COVID-19 Adult/YouNg InDividuals LOCKDOWN EXPERIENCE QUESTIONNAIRE
BMI	Body Mass Index
*df*	Degree of freedom
